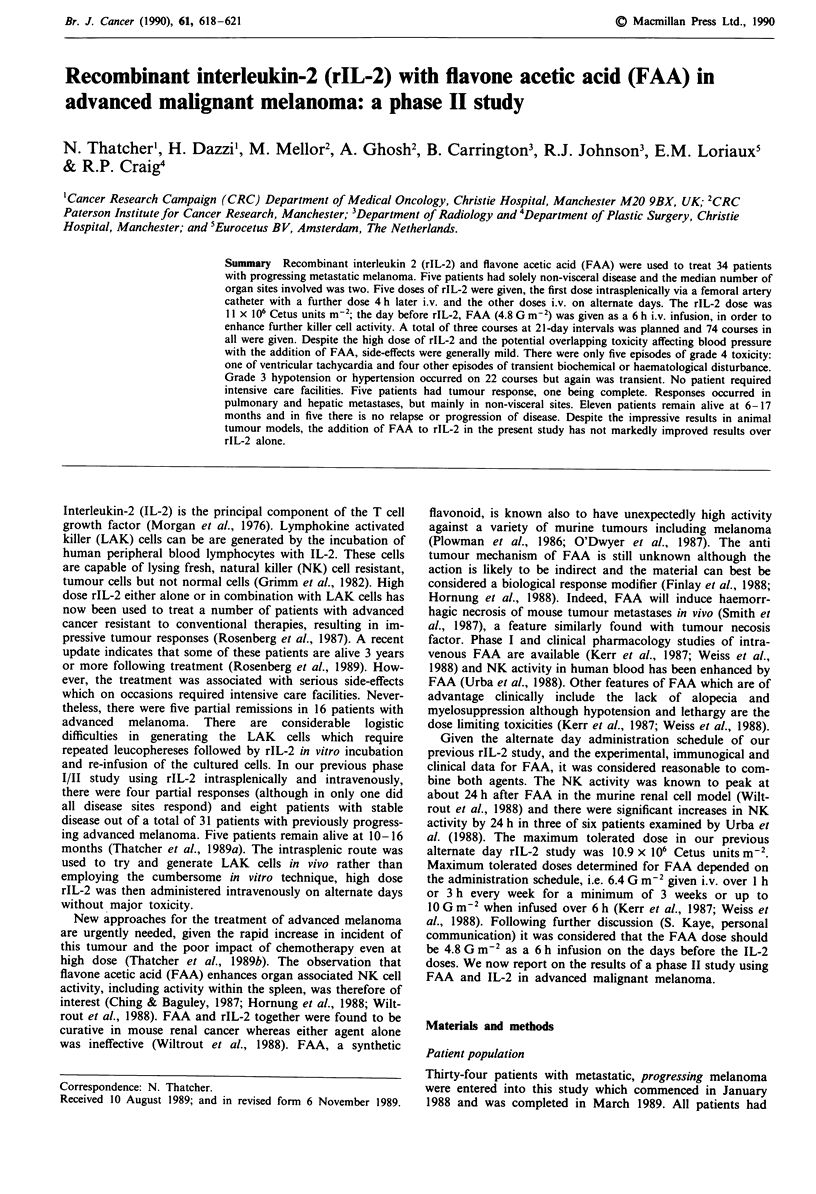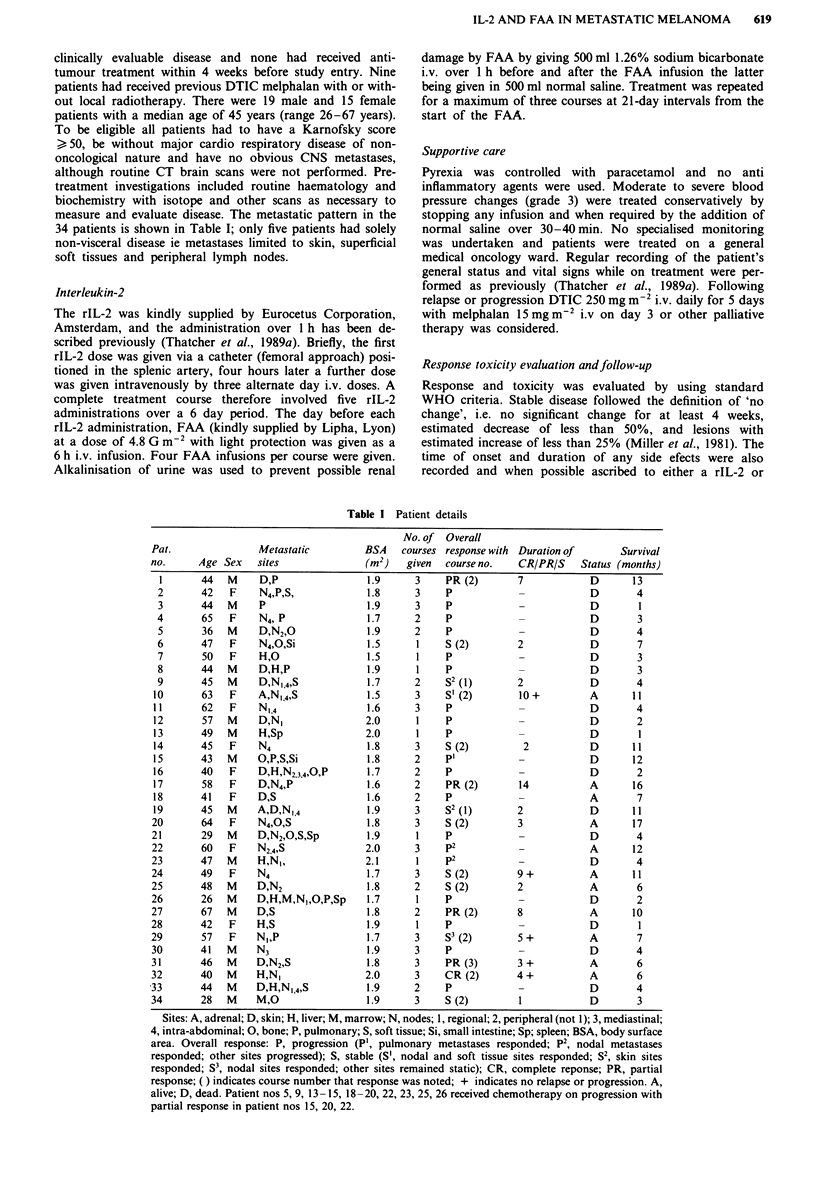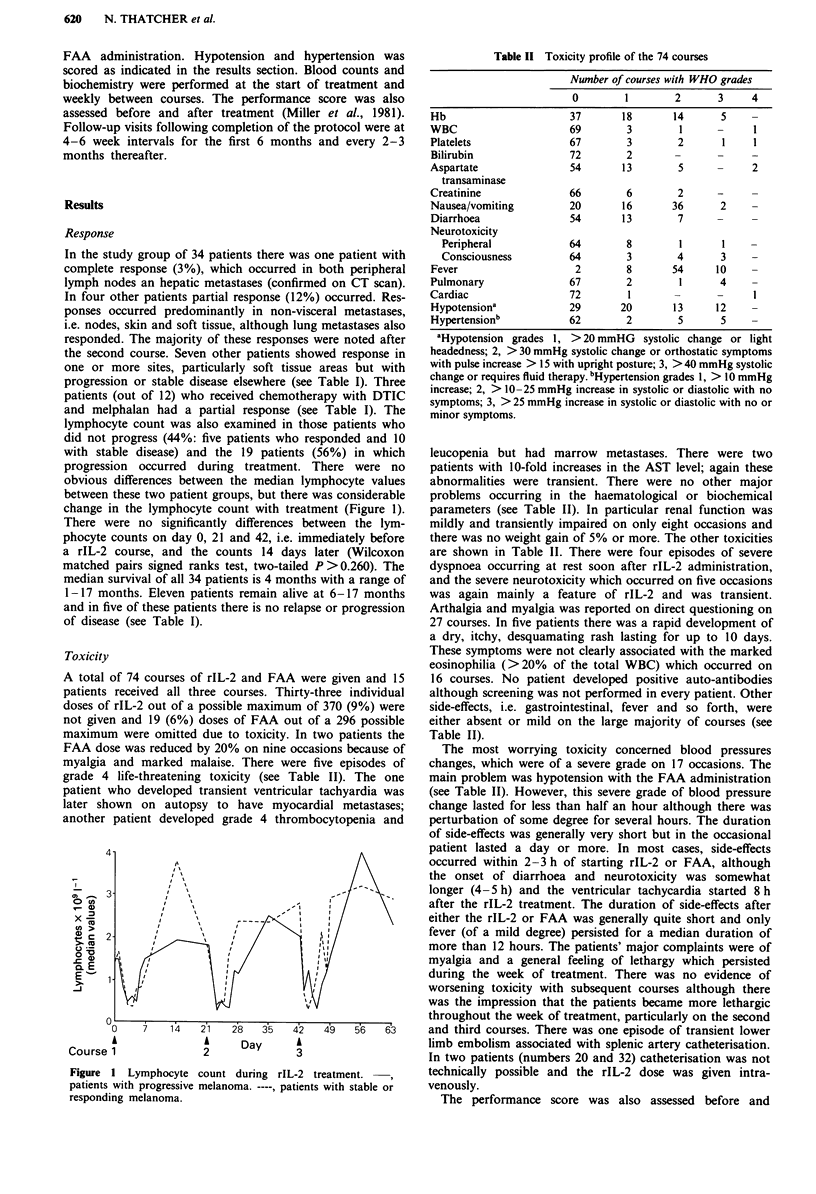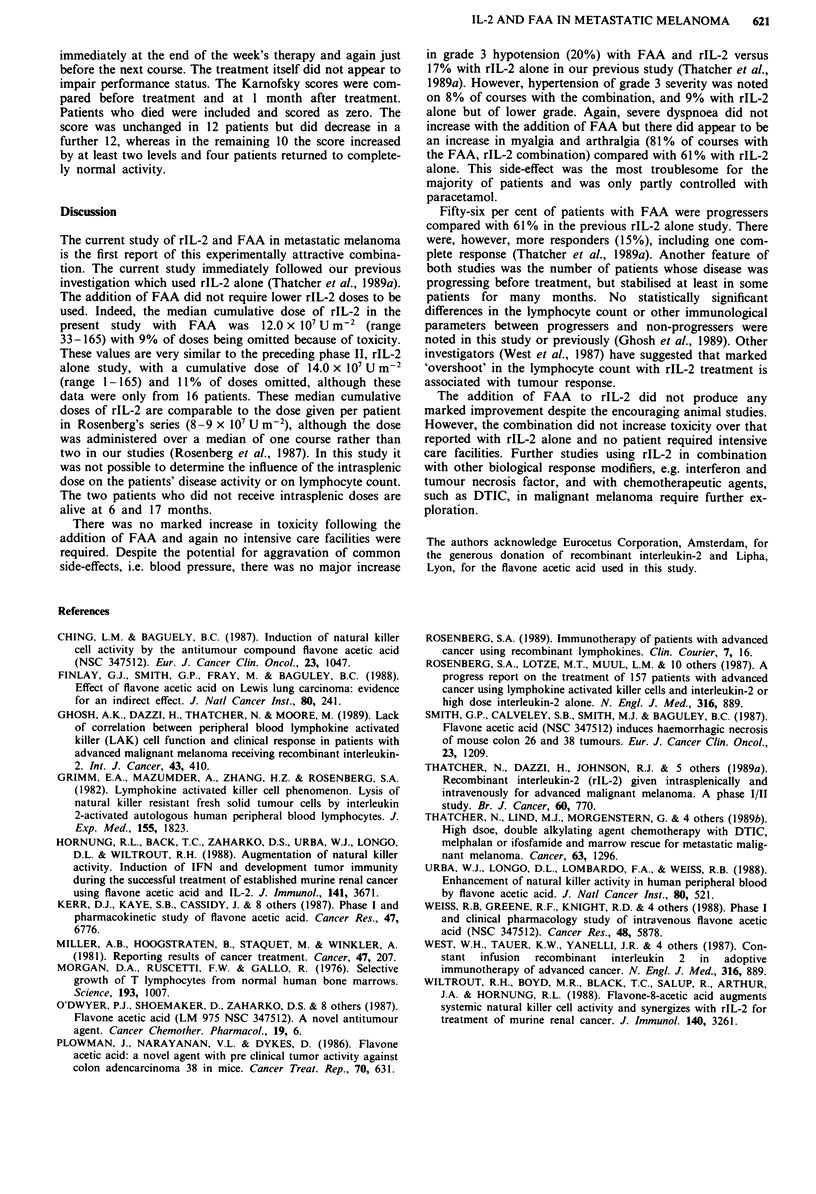# Recombinant interleukin-2 (rIL-2) with flavone acetic acid (FAA) in advanced malignant melanoma: a phase II study.

**DOI:** 10.1038/bjc.1990.137

**Published:** 1990-04

**Authors:** N. Thatcher, H. Dazzi, M. Mellor, A. Ghosh, B. Carrington, R. J. Johnson, E. M. Loriaux, R. P. Craig

**Affiliations:** Department of Medical Oncology, Christie Hospital, Manchester, UK.

## Abstract

Recombinant interleukin 2 (rIL-2) and flavone acetic acid (FAA) were used to treat 34 patients with progressing metastatic melanoma. Five patients had solely non-visceral disease and the median number of organ sites involved was two. Five doses of rIL-2 were given, the first dose intrasplenically via a femoral artery catheter with a further dose 4 h later i.v. and the other doses i.v. on alternate days. The rIL-2 dose was 11 x 10(6) Cetus units m-2; the day before rIL-2, FAA (4.8 G m-2) was given as a 6 h i.v. infusion, in order to enhance further killer cell activity. A total of three courses at 21-day intervals was planned and 74 courses in all were given. Despite the high dose of rIL-2 and the potential overlapping toxicity affecting blood pressure with the addition of FAA, side-effects were generally mild. There were only five episodes of grade 4 toxicity: one of ventricular tachycardia and four other episodes of transient biochemical or haematological disturbance. Grade 3 hypotension or hypertension occurred on 22 courses but again was transient. No patient required intensive care facilities. Five patients had tumour response, one being complete. Responses occurred in pulmonary and hepatic metastases, but mainly in non-visceral sites. Eleven patients remain alive at 6-17 months and in five there is no relapse or progression of disease. Despite the impressive results in animal tumour models, the addition of FAA to rIL-2 in the present study has not markedly improved results over rIL-2 alone.


Br. J. Cancer (1990), 61, 618-621 © Macmillan Press Ltd., 1990

### Recombinant interleukin-2 (rIL-2) with flavone acetic acid (FAA) in advanced malignant melanoma: a phase II study

N. Thatcher[1], H. Dazzi[1], M. Mellor[2], A. Ghosh[2], B. Carrington[3], R.J. Johnson[3], E.M. Loriaux[5] & R.P. Craig[4]

[1]*Cancer Research Campaign (CRC) Department of Medical Oncology, Christie Hospital, Manchester M20 9BX, UK;* [2]*CRC Paterson Institute for Cancer Research, Manchester;* [3]*Department of Radiology and* [4]*Department of Plastic Surgery, Christie Hospital, Manchester; and* [5]*Eurocetus BV, Amsterdam, The Netherlands.*

**Summary** Recombinant interleukin 2 (rIL-2) and flavone acetic acid (FAA) were used to treat 34 patients with progressing metastatic melanoma. Five patients had solely non-visceral disease and the median number of organ sites involved was two. Five doses of rIL-2 were given, the first dose intrasplenically via a femoral artery catheter with a further dose 4 h later i.v. and the other doses i.v. on alternate days. The rIL-2 dose was $11 \times 10^6$ Cetus units $m^{-2}$; the day before rIL-2, FAA (4.8 G $m^{-2}$) was given as a 6 h i.v. infusion, in order to enhance further killer cell activity. A total of three courses at 21-day intervals was planned and 74 courses in all were given. Despite the high dose of rIL-2 and the potential overlapping toxicity affecting blood pressure with the addition of FAA, side-effects were generally mild. There were only five episodes of grade 4 toxicity: one of ventricular tachycardia and four other episodes of transient biochemical or haematological disturbance. Grade 3 hypotension or hypertension occurred on 22 courses but again was transient. No patient required intensive care facilities. Five patients had tumour response, one being complete. Responses occurred in pulmonary and hepatic metastases, but mainly in non-visceral sites. Eleven patients remain alive at 6-17 months and in five there is no relapse or progression of disease. Despite the impressive results in animal tumour models, the addition of FAA to rIL-2 in the present study has not markedly improved results over rIL-2 alone.

Interleukin-2 (IL-2) is the principal component of the T cell growth factor (Morgan *et al.*, 1976). Lymphokine activated killer (LAK) cells can be are generated by the incubation of human peripheral blood lymphocytes with IL-2. These cells are capable of lysing fresh, natural killer (NK) cell resistant, tumour cells but not normal cells (Grimm *et al.*, 1982). High dose rIL-2 either alone or in combination with LAK cells has now been used to treat a number of patients with advanced cancer resistant to conventional therapies, resulting in impressive tumour responses (Rosenberg *et al.*, 1987). A recent update indicates that some of these patients are alive 3 years or more following treatment (Rosenberg *et al.*, 1989). However, the treatment was associated with serious side-effects which on occasions required intensive care facilities. Nevertheless, there were five partial remissions in 16 patients with advanced melanoma. There are considerable logistic difficulties in generating the LAK cells which require repeated leucophereses followed by rIL-2 *in vitro* incubation and re-infusion of the cultured cells. In our previous phase I/II study using rIL-2 intrasplenically and intravenously, there were four partial responses (although in only one did all disease sites respond) and eight patients with stable disease out of a total of 31 patients with previously progressing advanced melanoma. Five patients remain alive at 10-16 months (Thatcher *et al.*, 1989*a*). The intrasplenic route was used to try and generate LAK cells *in vivo* rather than employing the cumbersome *in vitro* technique, high dose rIL-2 was then administered intravenously on alternate days without major toxicity.

New approaches for the treatment of advanced melanoma are urgently needed, given the rapid increase in incident of this tumour and the poor impact of chemotherapy even at high dose (Thatcher *et al.*, 1989*b*). The observation that flavone acetic acid (FAA) enhances organ associated NK cell activity, including activity within the spleen, was therefore of interest (Ching & Baguley, 1987; Hornung *et al.*, 1988; Wiltrout *et al.*, 1988). FAA and rIL-2 together were found to be curative in mouse renal cancer whereas either agent alone was ineffective (Wiltrout *et al.*, 1988). FAA, a synthetic flavonoid, is known also to have unexpectedly high activity against a variety of murine tumours including melanoma (Plowman *et al.*, 1986; O'Dwyer *et al.*, 1987). The anti tumour mechanism of FAA is still unknown although the action is likely to be indirect and the material can best be considered a biological response modifier (Finlay *et al.*, 1988; Hornung *et al.*, 1988). Indeed, FAA will induce haemorrhagic necrosis of mouse tumour metastases *in vivo* (Smith *et al.*, 1987), a feature similarly found with tumour necosis factor. Phase I and clinical pharmacology studies of intravenous FAA are available (Kerr *et al.*, 1987; Weiss *et al.*, 1988) and NK activity in human blood has been enhanced by FAA (Urba *et al.*, 1988). Other features of FAA which are of advantage clinically include the lack of alopecia and myelosuppression although hypotension and lethargy are the dose limiting toxicities (Kerr *et al.*, 1987; Weiss *et al.*, 1988).

Given the alternate day administration schedule of our previous rIL-2 study, and the experimental, immunogical and clinical data for FAA, it was considered reasonable to combine both agents. The NK activity was known to peak at about 24 h after FAA in the murine renal cell model (Wiltrout *et al.*, 1988) and there were significant increases in NK activity by 24 h in three of six patients examined by Urba *et al.* (1988). The maximum tolerated dose in our previous alternate day rIL-2 study was $10.9 \times 10^6$ Cetus units $m^{-2}$. Maximum tolerated doses determined for FAA depended on the administration schedule, i.e. 6.4 G $m^{-2}$ given i.v. over 1 h or 3 h every week for a minimum of 3 weeks or up to 10 G $m^{-2}$ when infused over 6 h (Kerr *et al.*, 1987; Weiss *et al.*, 1988). Following further discussion (S. Kaye, personal communication) it was considered that the FAA dose should be 4.8 G $m^{-2}$ as a 6 h infusion on the days before the IL-2 doses. We now report on the results of a phase II study using FAA and IL-2 in advanced malignant melanoma.

### Materials and methods

### *Patient population*

Thirty-four patients with metastatic, *progressing* melanoma were entered into this study which commenced in January 1988 and was completed in March 1989. All patients had

Correspondence: N. Thatcher.
Received 10 August 1989; and in revised form 6 November 1989.

clinically evaluable disease and none had received anti-tumour treatment within 4 weeks before study entry. Nine patients had received previous DTIC melphalan with or without local radiotherapy. There were 19 male and 15 female patients with a median age of 45 years (range 26–67 years). To be eligible all patients had to have a Karnofsky score $\geqslant 50$, be without major cardio respiratory disease of non-oncological nature and have no obvious CNS metastases, although routine CT brain scans were not performed. Pre-treatment investigations included routine haematology and biochemistry with isotope and other scans as necessary to measure and evaluate disease. The metastatic pattern in the 34 patients is shown in Table I; only five patients had solely non-visceral disease ie metastases limited to skin, superficial soft tissues and peripheral lymph nodes.

*Interleukin-2*

The rIL-2 was kindly supplied by Eurocetus Corporation, Amsterdam, and the administration over 1 h has been described previously (Thatcher *et al.*, 1989*a*). Briefly, the first rIL-2 dose was given via a catheter (femoral approach) positioned in the splenic artery, four hours later a further dose was given intravenously by three alternate day i.v. doses. A complete treatment course therefore involved five rIL-2 administrations over a 6 day period. The day before each rIL-2 administration, FAA (kindly supplied by Lipha, Lyon) at a dose of 4.8 G $m^{-2}$ with light protection was given as a 6 h i.v. infusion. Four FAA infusions per course were given. Alkalinisation of urine was used to prevent possible renal damage by FAA by giving 500 ml 1.26% sodium bicarbonate i.v. over 1 h before and after the FAA infusion the latter being given in 500 ml normal saline. Treatment was repeated for a maximum of three courses at 21-day intervals from the start of the FAA.

*Supportive care*

Pyrexia was controlled with paracetamol and no anti inflammatory agents were used. Moderate to severe blood pressure changes (grade 3) were treated conservatively by stopping any infusion and when required by the addition of normal saline over 30–40 min. No specialised monitoring was undertaken and patients were treated on a general medical oncology ward. Regular recording of the patient's general status and vital signs while on treatment were performed as previously (Thatcher *et al.*, 1989*a*). Following relapse or progression DTIC 250 mg $m^{-2}$ i.v. daily for 5 days with melphalan 15 mg $m^{-2}$ i.v on day 3 or other palliative therapy was considered.

*Response toxicity evaluation and follow-up*

Response and toxicity was evaluated by using standard WHO criteria. Stable disease followed the definition of 'no change', i.e. no significant change for at least 4 weeks, estimated decrease of less than 50%, and lesions with estimated increase of less than 25% (Miller *et al.*, 1981). The time of onset and duration of any side efects were also recorded and when possible ascribed to either a rIL-2 or

**Table I** Patient details

| *Pat. no.* | *Age* | *Sex* | *Metastatic sites* | *BSA ($m^2$)* | *No. of courses given* | *Overall response with course no.* | *Duration of CR/PR/S* | *Status* | *Survival (months)* |
|---|---|---|---|---|---|---|---|---|---|
| 1 | 44 | M | D,P | 1.9 | 3 | PR (2) | 7 | D | 13 |
| 2 | 42 | F | $N_4$,P,S, | 1.8 | 3 | P | – | D | 4 |
| 3 | 44 | M | P | 1.9 | 3 | P | – | D | 1 |
| 4 | 65 | F | $N_4$, P | 1.7 | 2 | P | – | D | 3 |
| 5 | 36 | M | D,$N_2$,O | 1.9 | 2 | P | – | D | 4 |
| 6 | 47 | F | $N_4$,O,Si | 1.5 | 1 | S (2) | 2 | D | 7 |
| 7 | 50 | F | H,O | 1.5 | 1 | P | – | D | 3 |
| 8 | 44 | M | D,H,P | 1.9 | 1 | P | – | D | 3 |
| 9 | 45 | M | D,$N_{1,4}$,S | 1.7 | 2 | $S^2$ (1) | 2 | D | 4 |
| 10 | 63 | F | A,$N_{1,4}$,S | 1.5 | 3 | $S^1$ (2) | 10+ | A | 11 |
| 11 | 62 | F | $N_{1,4}$ | 1.6 | 3 | P | – | D | 4 |
| 12 | 57 | M | D,$N_1$ | 2.0 | 1 | P | – | D | 2 |
| 13 | 49 | M | H,Sp | 2.0 | 1 | P | – | D | 1 |
| 14 | 45 | F | $N_4$ | 1.8 | 3 | S (2) | 2 | D | 11 |
| 15 | 43 | M | O,P,S,Si | 1.8 | 2 | $P^1$ | – | D | 12 |
| 16 | 40 | F | D,H,$N_{2,3,4}$,O,P | 1.7 | 2 | P | – | D | 2 |
| 17 | 58 | F | D,$N_4$,P | 1.6 | 2 | PR (2) | 14 | A | 16 |
| 18 | 41 | F | D,S | 1.6 | 2 | P | – | A | 7 |
| 19 | 45 | M | A,D,$N_{1,4}$ | 1.9 | 3 | $S^2$ (1) | 2 | D | 11 |
| 20 | 64 | F | $N_4$,O,S | 1.8 | 3 | S (2) | 3 | A | 17 |
| 21 | 29 | M | D,$N_2$,O,S,Sp | 1.9 | 1 | P | – | D | 4 |
| 22 | 60 | F | $N_{2,4}$,S | 2.0 | 3 | $P^2$ | – | A | 12 |
| 23 | 47 | M | H,$N_1$, | 2.1 | 1 | $P^2$ | – | D | 4 |
| 24 | 49 | F | $N_4$ | 1.7 | 3 | S (2) | 9+ | A | 11 |
| 25 | 48 | M | D,$N_2$ | 1.8 | 2 | S (2) | 2 | A | 6 |
| 26 | 26 | M | D,H,M,$N_1$,O,P,Sp | 1.7 | 1 | P | – | D | 2 |
| 27 | 67 | M | D,S | 1.8 | 2 | PR (2) | 8 | A | 10 |
| 28 | 42 | F | H,S | 1.9 | 1 | P | – | D | 1 |
| 29 | 57 | F | $N_1$,P | 1.7 | 3 | $S^3$ (2) | 5+ | A | 7 |
| 30 | 41 | M | $N_3$ | 1.9 | 3 | P | – | D | 4 |
| 31 | 46 | M | D,$N_2$,S | 1.8 | 3 | PR (3) | 3+ | A | 6 |
| 32 | 40 | M | H,$N_1$ | 2.0 | 3 | CR (2) | 4+ | A | 6 |
| 33 | 44 | M | D,H,$N_{1,4}$,S | 1.9 | 2 | P | – | D | 4 |
| 34 | 28 | M | M,O | 1.9 | 3 | S (2) | 1 | D | 3 |

Sites: A, adrenal; D, skin; H, liver; M, marrow; N, nodes; 1, regional; 2, peripheral (not 1); 3, mediastinal; 4, intra-abdominal; O, bone; P, pulmonary; S, soft tissue; Si, small intestine; Sp; spleen; BSA, body surface area. Overall response: P, progression ($P^1$, pulmonary metastases responded; $P^2$, nodal metastases responded; other sites progressed); S, stable ($S^1$, nodal and soft tissue sites responded; $S^2$, skin sites responded; $S^3$, nodal sites responded; other sites remained static); CR, complete reponse; PR, partial response; ( ) indicates course number that response was noted; + indicates no relapse or progression. A, alive; D, dead. Patient nos 5, 9, 13–15, 18–20, 22, 23, 25, 26 received chemotherapy on progression with partial response in patient nos 15, 20, 22.

FAA administration. Hypotension and hypertension was scored as indicated in the results section. Blood counts and biochemistry were performed at the start of treatment and weekly between courses. The performance score was also assessed before and after treatment (Miller *et al.*, 1981). Follow-up visits following completion of the protocol were at 4–6 week intervals for the first 6 months and every 2–3 months thereafter.

### Results

### *Response*

In the study group of 34 patients there was one patient with complete response (3%), which occurred in both peripheral lymph nodes an hepatic metastases (confirmed on CT scan). In four other patients partial response (12%) occurred. Responses occurred predominantly in non-visceral metastases, i.e. nodes, skin and soft tissue, although lung metastases also responded. The majority of these responses were noted after the second course. Seven other patients showed response in one or more sites, particularly soft tissue areas but with progression or stable disease elsewhere (see Table I). Three patients (out of 12) who received chemotherapy with DTIC and melphalan had a partial response (see Table I). The lymphocyte count was also examined in those patients who did not progress (44%: five patients who responded and 10 with stable disease) and the 19 patients (56%) in which progression occurred during treatment. There were no obvious differences between the median lymphocyte values between these two patient groups, but there was considerable change in the lymphocyte count with treatment (Figure 1). There were no significantly differences between the lymphocyte counts on day 0, 21 and 42, i.e. immediately before a rIL-2 course, and the counts 14 days later (Wilcoxon matched pairs signed ranks test, two-tailed $P > 0.260$). The median survival of all 34 patients is 4 months with a range of 1–17 months. Eleven patients remain alive at 6–17 months and in five of these patients there is no relapse or progression of disease (see Table I).

### *Toxicity*

A total of 74 courses of rIL-2 and FAA were given and 15 patients received all three courses. Thirty-three individual doses of rIL-2 out of a possible maximum of 370 (9%) were not given and 19 (6%) doses of FAA out of a 296 possible maximum were omitted due to toxicity. In two patients the FAA dose was reduced by 20% on nine occasions because of myalgia and marked malaise. There were five episodes of grade 4 life-threatening toxicity (see Table II). The one patient who developed transient ventricular tachyardia was later shown on autopsy to have myocardial metastases; another patient developed grade 4 thrombocytopenia and leucopenia but had marrow metastases. There were two patients with 10-fold increases in the AST level; again these abnormalities were transient. There were no other major problems occurring in the haematological or biochemical parameters (see Table II). In particular renal function was mildly and transiently impaired on only eight occasions and there was no weight gain of 5% or more. The other toxicities are shown in Table II. There were four episodes of severe dyspnoea occurring at rest soon after rIL-2 administration, and the severe neurotoxicity which occurred on five occasions was again mainly a feature of rIL-2 and was transient. Arthalgia and myalgia was reported on direct questioning on 27 courses. In five patients there was a rapid development of a dry, itchy, desquamating rash lasting for up to 10 days. These symptoms were not clearly associated with the marked eosinophilia (>20% of the total WBC) which occurred on 16 courses. No patient developed positive auto-antibodies although screening was not performed in every patient. Other side-effects, i.e. gastrointestinal, fever and so forth, were either absent or mild on the large majority of courses (see Table II).

**Figure 1** Lymphocyte count during rIL-2 treatment. —, patients with progressive melanoma. ----, patients with stable or responding melanoma.

**Table II** Toxicity profile of the 74 courses

| | *Number of courses with WHO grades* | | | | |
|---|---|---|---|---|---|
| | 0 | 1 | 2 | 3 | 4 |
| Hb | 37 | 18 | 14 | 5 | – |
| WBC | 69 | 3 | 1 | – | 1 |
| Platelets | 67 | 3 | 2 | 1 | 1 |
| Bilirubin | 72 | 2 | – | – | – |
| Aspartate transaminase | 54 | 13 | 5 | – | 2 |
| Creatinine | 66 | 6 | 2 | – | – |
| Nausea/vomiting | 20 | 16 | 36 | 2 | – |
| Diarrhoea | 54 | 13 | 7 | – | – |
| Neurotoxicity | | | | | |
| Peripheral | 64 | 8 | 1 | 1 | – |
| Consciousness | 64 | 3 | 4 | 3 | – |
| Fever | 2 | 8 | 54 | 10 | – |
| Pulmonary | 67 | 2 | 1 | 4 | – |
| Cardiac | 72 | 1 | – | – | 1 |
| Hypotension[a] | 29 | 20 | 13 | 12 | – |
| Hypertension[b] | 62 | 2 | 5 | 5 | – |

[a]Hypotension grades 1, >20 mmHG systolic change or light headedness; 2, >30 mmHg systolic change or orthostatic symptoms with pulse increase >15 with upright posture; 3, >40 mmHg systolic change or requires fluid therapy. [b]Hypertension grades 1, >10 mmHg increase; 2, >10–25 mmHg increase in systolic or diastolic with no symptoms; 3, >25 mmHg increase in systolic or diastolic with no or minor symptoms.

The most worrying toxicity concerned blood pressures changes, which were of a severe grade on 17 occasions. The main problem was hypotension with the FAA administration (see Table II). However, this severe grade of blood pressure change lasted for less than half an hour although there was perturbation of some degree for several hours. The duration of side-effects was generally very short but in the occasional patient lasted a day or more. In most cases, side-effects occurred within 2–3 h of starting rIL-2 or FAA, although the onset of diarrhoea and neurotoxicity was somewhat longer (4–5 h) and the ventricular tachycardia started 8 h after the rIL-2 treatment. The duration of side-effects after either the rIL-2 or FAA was generally quite short and only fever (of a mild degree) persisted for a median duration of more than 12 hours. The patients' major complaints were of myalgia and a general feeling of lethargy which persisted during the week of treatment. There was no evidence of worsening toxicity with subsequent courses although there was the impression that the patients became more lethargic throughout the week of treatment, particularly on the second and third courses. There was one episode of transient lower limb embolism associated with splenic artery catheterisation. In two patients (numbers 20 and 32) catheterisation was not technically possible and the rIL-2 dose was given intravenously.

The performance score was also assessed before and immediately at the end of the week's therapy and again just before the next course. The treatment itself did not appear to impair performance status. The Karnofsky scores were compared before treatment and at 1 month after treatment. Patients who died were included and scored as zero. The score was unchanged in 12 patients but did decrease in a further 12, whereas in the remaining 10 the score increased by at least two levels and four patients returned to completely normal activity.

### Discussion

The current study of rIL-2 and FAA in metastatic melanoma is the first report of this experimentally attractive combination. The current study immediately followed our previous investigation which used rIL-2 alone (Thatcher *et al.*, 1989*a*). The addition of FAA did not require lower rIL-2 doses to be used. Indeed, the median cumulative dose of rIL-2 in the present study with FAA was $12.0 \times 10^7 \mathrm{U\,m^{-2}}$ (range 33–165) with 9% of doses being omitted because of toxicity. These values are very similar to the preceding phase II, rIL-2 alone study, with a cumulative dose of $14.0 \times 10^7 \mathrm{U\,m^{-2}}$ (range 1–165) and 11% of doses omitted, although these data were only from 16 patients. These median cumulative doses of rIL-2 are comparable to the dose given per patient in Rosenberg's series ($8-9 \times 10^7 \mathrm{U\,m^{-2}}$), although the dose was administered over a median of one course rather than two in our studies (Rosenberg *et al.*, 1987). In this study it was not possible to determine the influence of the intrasplenic dose on the patients' disease activity or on lymphocyte count. The two patients who did not receive intrasplenic doses are alive at 6 and 17 months.

There was no marked increase in toxicity following the addition of FAA and again no intensive care facilities were required. Despite the potential for aggravation of common side-effects, i.e. blood pressure, there was no major increase in grade 3 hypotension (20%) with FAA and rIL-2 versus 17% with rIL-2 alone in our previous study (Thatcher *et al.*, 1989*a*). However, hypertension of grade 3 severity was noted on 8% of courses with the combination, and 9% with rIL-2 alone but of lower grade. Again, severe dyspnoea did not increase with the addition of FAA but there did appear to be an increase in myalgia and arthralgia (81% of courses with the FAA, rIL-2 combination) compared with 61% with rIL-2 alone. This side-effect was the most troublesome for the majority of patients and was only partly controlled with paracetamol.

Fifty-six per cent of patients with FAA were progressers compared with 61% in the previous rIL-2 alone study. There were, however, more responders (15%), including one complete response (Thatcher *et al.*, 1989*a*). Another feature of both studies was the number of patients whose disease was progressing before treatment, but stabilised at least in some patients for many months. No statistically significant differences in the lymphocyte count or other immunological parameters between progressers and non-progressers were noted in this study or previously (Ghosh *et al.*, 1989). Other investigators (West *et al.*, 1987) have suggested that marked 'overshoot' in the lymphocyte count with rIL-2 treatment is associated with tumour response.

The addition of FAA to rIL-2 did not produce any marked improvement despite the encouraging animal studies. However, the combination did not increase toxicity over that reported with rIL-2 alone and no patient required intensive care facilities. Further studies using rIL-2 in combination with other biological response modifiers, e.g. interferon and tumour necrosis factor, and with chemotherapeutic agents, such as DTIC, in malignant melanoma require further exploration.

The authors acknowledge Eurocetus Corporation, Amsterdam, for the generous donation of recombinant interleukin-2 and Lipha, Lyon, for the flavone acetic acid used in this study.

### References

CHING, L.M. & BAGUELY, B.C. (1987). Induction of natural killer cell activity by the antitumour compound flavone acetic acid (NSC 347512). *Eur. J. Cancer Clin. Oncol.*, **23**, 1047.

FINLAY, G.J., SMITH, G.P., FRAY, M. & BAGULEY, B.C. (1988). Effect of flavone acetic acid on Lewis lung carcinoma: evidence for an indirect effect. *J. Natl Cancer Inst.*, **80**, 241.

GHOSH, A.K., DAZZI, H., THATCHER, N. & MOORE, M. (1989). Lack of correlation between peripheral blood lymphokine activated killer (LAK) cell function and clinical response in patients with advanced malignant melanoma receiving recombinant interleukin-2. *Int. J. Cancer*, **43**, 410.

GRIMM, E.A., MAZUMDER, A., ZHANG, H.Z. & ROSENBERG, S.A. (1982). Lymphokine activated killer cell phenomenon. Lysis of natural killer resistant fresh solid tumour cells by interleukin 2-activated autologous human peripheral blood lymphocytes. *J. Exp. Med.*, **155**, 1823.

HORNUNG, R.L., BACK, T.C., ZAHARKO, D.S., URBA, W.J., LONGO, D.L. & WILTROUT, R.H. (1988). Augmentation of natural killer activity. Induction of IFN and development tumor immunity during the successful treatment of established murine renal cancer using flavone acetic acid and IL-2. *J. Immunol.*, **141**, 3671.

KERR, D.J., KAYE, S.B., CASSIDY, J. & 8 others (1987). Phase I and pharmacokinetic study of flavone acetic acid. *Cancer Res.*, **47**, 6776.

MILLER, A.B., HOOGSTRATEN, B., STAQUET, M. & WINKLER, A. (1981). Reporting results of cancer treatment. *Cancer*, **47**, 207.

MORGAN, D.A., RUSCETTI, F.W. & GALLO, R. (1976). Selective growth of T lymphocytes from normal human bone marrows. *Science*, **193**, 1007.

O'DWYER, P.J., SHOEMAKER, D., ZAHARKO, D.S. & 8 others (1987). Flavone acetic acid (LM 975 NSC 347512). A novel antitumour agent. *Cancer Chemother. Pharmacol.*, **19**, 6.

PLOWMAN, J., NARAYANAN, V.L. & DYKES, D. (1986). Flavone acetic acid: a novel agent with pre clinical tumor activity against colon adencarcinoma 38 in mice. *Cancer Treat. Rep.*, **70**, 631.

ROSENBERG, S.A. (1989). Immunotherapy of patients with advanced cancer using recombinant lymphokines. *Clin. Courier*, **7**, 16.

ROSENBERG, S.A., LOTZE, M.T., MUUL, L.M. & 10 others (1987). A progress report on the treatment of 157 patients with advanced cancer using lymphokine activated killer cells and interleukin-2 or high dose interleukin-2 alone. *N. Engl. J. Med.*, **316**, 889.

SMITH, G.P., CALVELEY, S.B., SMITH, M.J. & BAGULEY, B.C. (1987). Flavone acetic acid (NSC 347512) induces haemorrhagic necrosis of mouse colon 26 and 38 tumours. *Eur. J. Cancer Clin. Oncol.*, **23**, 1209.

THATCHER, N., DAZZI, H., JOHNSON, R.J. & 5 others (1989*a*). Recombinant interleukin-2 (rIL-2) given intrasplenically and intravenously for advanced malignant melanoma. A phase I/II study. *Br. J. Cancer*, **60**, 770.

THATCHER, N., LIND, M.J., MORGENSTERN, G. & 4 others (1989*b*). High dsoe, double alkylating agent chemotherapy with DTIC, melphalan or ifosfamide and marrow rescue for metastatic malignant melanoma. *Cancer*, **63**, 1296.

URBA, W.J., LONGO, D.L., LOMBARDO, F.A., & WEISS, R.B. (1988). Enhancement of natural killer activity in human peripheral blood by flavone acetic acid. *J. Natl Cancer Inst.*, **80**, 521.

WEISS, R.B, GREENE, R.F., KNIGHT, R.D. & 4 others (1988). Phase I and clinical pharmacology study of intravenous flavone acetic acid (NSC 347512). *Cancer Res.*, **48**, 5878.

WEST, W.H., TAUER, K.W., YANELLI, J.R. & 4 others (1987). Constant infusion recombinant interleukin 2 in adoptive immunotherapy of advanced cancer. *N. Engl. J. Med.*, **316**, 889.

WILTROUT, R.H., BOYD, M.R., BLACK, T.C., SALUP, R., ARTHUR, J.A. & HORNUNG, R.L. (1988). Flavone-8-acetic acid augments systemic natural killer cell activity and synergizes with rIL-2 for treatment of murine renal cancer. *J. Immunol.* **140**, 3261.